# LC/MS analysis of mushrooms provided new insights into dietary management of diabetes mellitus in rats

**DOI:** 10.1002/fsn3.3236

**Published:** 2023-01-27

**Authors:** Abdelaziz Hussein, Abdallah Ghonimy, Hailong Jiang, Guixin Qin, Saeed El‐Ashram, Saddam Hussein, Ibrahim Abd El‐Razek, Tarek El‐Afifi, Mohammed Hamdy Farouk

**Affiliations:** ^1^ College of Animal Science and Technology Jilin Agricultural University Changchun China; ^2^ Jilin Provincial Key Lab of Animal Nutrition and Feed Science Jilin Agricultural University Changchun China; ^3^ Regional Center for Food and Feed Agricultural Research Center Giza Egypt; ^4^ Fish Farming and Technology Institute Suez Canal University Ismailia Egypt; ^5^ Key Laboratory of Sustainable Development of Marine Fisheries, Yellow Sea Fisheries Research Institute Chinese Academy of Fishery Sciences Qingdao China; ^6^ Laboratory for Marine Fisheries Science and Food Production Processes Qingdao National Laboratory for Marine Science and Technology Qingdao China; ^7^ School of Life Science and Engineering Foshan University Foshan China; ^8^ Faculty of Science Kafrelsheikh University Kafr El‐Sheikh Egypt; ^9^ Animal Production Department, Faculty of Agriculture Kafrelsheikh University Kafr El‐Sheikh Egypt; ^10^ Animal Production Department, Faculty of Agriculture Al‐Azhar University Cairo Egypt

**Keywords:** 16S rRNA, alloxan, diabetes, dietary fiber, microbiota, mushroom

## Abstract

Mushrooms possess antihyperglycemic effect on diabetic individuals due to their nonfibrous and fibrous bioactive compounds. This study aimed to reveal the effect of different types of mushrooms on plasma glucose level and gut microbiota composition in diabetic individuals. The effects of five different mushroom species (*Ganoderma lucidum*, GLM; *Pleurotus ostreatus*, POM; *Pleurotus citrinopileatus*, PCM; *Lentinus edodes*, LEM; or *Hypsizigus marmoreus*, HMM) on alloxan‐induced diabetic rats were investigated in this study. The results indicated that LEM and HMM treatments showed lower plasma glucose levels. For the microbiota composition, *ACE*, *Chao1*, *Shannon*, and *Simpson* were significantly affected by PCM and LEM treatments (*p <* .05), while ACE, Shannon, and Simpson indexes were affected by HMM treatment (*p <* .01). Simpson index was affected in positive control (C+) and POM groups. All these four indices were lower in GLM treatment (*p <* .05). Dietary supplementation of mushrooms reduced plasma glucose level directly through mushrooms' bioactive compounds (agmatine, sphingosine, pyridoxine, linolenic, and alanine) and indirectly through stachyose (oligosaccharide) and gut microbiota modulation. In conclusion, LEM and HMM can be used as food additives to improve plasma glucose level and gut microbiome composition in diabetic individuals.

## Graphical abstract

The effect of mushroom's bioactive compounds on plasma glucose level. Agmatine increases insulin secretion and glucose uptake in muscle. Sphingosine activates omega‐3 fatty acid receptor (GPR120) mediating potent insulin‐sensitizing effects. Pyridoxine decreases insulin resistance. Stachyose regulates the intestinal microflora balance.

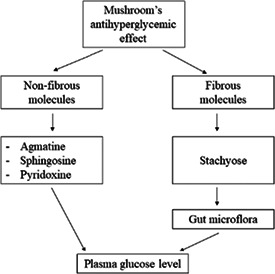

## 1 INTRODUCTION

Mushrooms possess a necessary dietary ingredient due to their low‐calorie value, different bioactive compounds, and rich fiber content. These compounds include vitamins (such as riboflavin and niacin), minerals (such as iron and phosphorus) (Alkin et al., 2021), and fibers (Lu et al., 2020). Moreover, mushrooms differ in their content of phenols (Alkin et al., 2021) and antioxidants (ergothioneine and glutathione) (Beelman et al., 2019). Thus, each mushroom type possesses different content of bioactive compounds, fiber contents, or both (Alkin et al., 2021). Gut microbiota degrades the dietary fibers as a nondigestible carbon sources and change the microbiota composition (Tang et al., 2017). Unfavorable modulations of gut microbiota are associated with multiple chronic diseases including diabetes mellitus (Tang et al., 2017). In particular, mushrooms possess enriched fibers, such as polysaccharides and heteroglucans (Li et al., 2021), these decrease a pathogen proliferation by inducing the growth of probiotic bacteria in gut (Kumari, 2020). For instance, the species of *Ganoderm lucidum* is a medicinal mushroom which contains various bioactive compounds that operates as antimicrobial agents (Cor et al., 2018). Fruiting bodies and mycelia of *G. lucidum* contain polysaccharides, such as glycoproteins, (1 → 3), (1 → 6)‐a/β‐glucans, and water‐soluble heteropolysaccharides (Martin & Jiang, 2010). Those polysaccharides have antihypoglycemic effect (Chassaing et al., 2017; Xu et al., 2017). *G. lucidum* increases anti‐inflammatory bacteria (*Enterococcus* and *Dehalobacterium*) in mice diabetic individuals (Chen, Liu, et al., 2020; Chen, Xiao, et al., 2020) and decreases the abundance of harmful bacteria, such as *Aerococcus*, *Corynebactrium*, *Ruminococcus*, and *Proteus* in type‐2 mice diabetic groups (Chen, Liu, et al., 2020; Chen, Xiao, et al., 2020). *G. lucidum* treatment on type‐2 diabetes, enhanced SCFA‐producing bacterial activity (Chen, Xiao, et al., 2020). In fact, oral administration of extracted polysaccharides from mushrooms (*Pleurotus eryngii* and *Poria cocos*) promote SCFA‐producing bacterial growth (Li et al., 2021), this improve energy metabolism through affecting the intestinal gluconeogenesis (IGN) and insulin sensitivity simulation (De Vadder et al., 2014). Since IGN releases glucose molecules which can be detected by the glucose sensor in the portal vein. Such signal is transmitted to the brain by the peripheral nervous system regulating glucose metabolism (Delaere et al., 2013). Polysaccharides inhibit digestive enzymes' activity based on the interaction with different sites at enzymes' structure. In addition, polysaccharide's viscoelasticity effect interferences with the enzymes and substrates flow resulting in lipid‐lowering effect (Xie et al., 2022).

Most mushroom‐related studies have investigated the effect of mushroom fibrous bioactive compounds on individual diabetics. While few studies have investigated the non‐fibrous bioactive compounds on diabetic individuals (Cor et al., 2018; Lu et al., 2020). Dubey et al. (2019) indicated that few bioactive compounds of edible mushrooms are identified. In a pilot study, mushroom untargeted molecules' composition was investigated, and analyzed; this revealed the existence of some of bioactive compounds in the tested mushrooms. The literature showed that agmatine has an antihyperglycemic effect through increasing glucose uptake in muscles by simulating insulin secretion (Chang et al., 2010; Malaisse et al., 1989; Naoki & Fujiwara, 2019; Nissim et al., 2006, 2014; Shepherd et al., 2012; Su et al., 2008, 2009). Whereas stachyose decreases the blood glucose level in alloxan‐induced diabetic rats (Zhang et al., 2004), in addition to its regulation effect on the intestinal microflora balance (Liu, Jia, et al., 2018; Liu, Wang, et al., 2018). Sphingosine (PHS) activates omega‐3 fatty acid receptor (GPR120) resulting in an insulin‐sensitizing effect (Rudd et al., 2020). Finally, pyridoxine decreases insulin resistance via scavenging the pathogenic reactive carbonyl species (Haus & Thyfault, 2018). There are epidemiological evidences supporting the non‐fibrous bioactive compounds safety use, eight mushrooms have been investigated for their effect on DM, that *G. lucidum* has showed the highest content of phenolic and flavonoids compounds (Wu & Xu, 2015). But this study did not analyze the other bioactive molecules. Generally, the non‐fibrous compounds are naturally existed in human foods, for example, agmatine in fermented foods (Galgano et al., 2012), stachyose (oligosaccharides) as a probiotic in human foods (Yang et al., 2018), sphingosine in dairy products (Possemiers et al., 2005), and pyridoxine is a vitamin B6 (Shaik Mohamed, 2001). This revealed a possible therapeutic potency of mushrooms on diabetic individuals.

Mushrooms are basic food components in Chinese table. Thus, revealing their nutritional value and their therapeutic potency are expected to increase the social awareness about dietary mushroom in addition to their therapeutic effect leading to the improving of public health.

To reveal the effect of those bioactive compounds in mushrooms on a diabetic individual, alloxan injection was used to induce the diabetes type II in rats by damaging pancreatic cells and initiating hyperglycemia (Inalegwu et al., 2021). These animals were subjected to different dietary mushrooms to reveal the effect of bioactive compounds sourced from mushrooms on blood glucose level and intestinal microbial composition as basic indicators for the possible effect.

We hypothesized that dietary mushrooms inclusion may decrease plasma glucose level and modulate microbiota composition directly via their bioactive molecules, and indirectly via fiber content and microflora modulation. This study aimed to reveal the effect of non‐fibrous bioactive compounds sourced from mushrooms on blood glucose level and intestinal microbial composition in diabetic rat individuals. These molecules may develop a food additive with an effective and specific health functionality considering food processing conditions and their effect on food quality.

## 2 MATERIALS AND METHODS

### 2.1 Experimental ethics and tested animals

This study was conducted under the ethical approval of Animal Welfare and Ethics Committee of the Key Laboratory of Animal Safety Production of Ministry of Education, PR China (no. KT2018011).

Diabetes mellitus is a metabolic disease characterized by hyperglycemia, occurring due to abnormal insulin action or insulin secretion. Alloxan induces diabetes type II by damaging pancreatic cells and initiating hyperglycemia (Inalegwu et al., 2021). Experimental animals Albino male rats (*Rattus norvegicus*) weighed 160–180 g with age 30 days were obtained from the animal's research center, Jilin Agricultural University, Changchun, China. Alloxan‐induced diabetic rats (AIDRs) were used as the diabetic model after acclimation period of 30 days. AIDRs were induced by intraperitoneal injection of Alloxan (150 mg/kg of body weight; Shanghai Sinyu Biotechnology Company) after an overnight fast. Three days after Alloxan injection, rats with a plasma glucose concentration of 11 mmol/L or above and symptoms of polyuria, polyphagia, and polydipsia were considered to have diabetes. The animals were distributed among treatments, seven animals per treatment (Table 1). All experimental animals were weighed weekly using a digital balance (Yeng Heng Electronic Scale Company) within the experimental period (4 weeks).

**TABLE 1:** Experimental design

Groups	Treatments	Diet (%)	No. of rats (*n*)
A	C−	100% Commercial diet	7
B	C+	100% Commercial diet	5
C	GLM	75% Commercial diet + 25% GLM	5
D	POM	75% Commercial diet + 25% POM	5
E	PCM	75% Commercial diet + 25% PCM	4
F	LEM	75% Commercial diet + 25% LEM	5
G	HMM	75% Commercial diet + 25% HMM	5

Abbreviations: C−, negative control; C+, positive control; GLM, *Ganoderma lucidum* mushroom; HMM, *Hypsizigus marmoreus* mushroom; LEM, *Lentinus edodes* mushroom; PCM, *Pleurotus citrinopileatus* mushroom; POM, *Pleurotus ostreatus* mushroom.

### 2.2 Determination of plasma glucose

Animals were deprived of food and water overnight and a blood glucose meter and test strips (Hangzhou Econ Biotech Company) were used to measure the blood glucose levels.

### 2.3 Experimental diets and mushrooms' bioactive compounds

The effect of five types of mushrooms (GLM) *Ganoderma lucidum* mushroom (traditional Chinese medicinal mushroom), (POM) *Pleurotus ostreatus* mushroom, (PCM) *Pleurotus citrinopileatus* mushroom, (LEM) *Lentinus edodes* mushroom, and (HMM) *Hypsizigus marmoreus* mushrooms were tested in rats that were fed on a commercial diet (Beijing Keao Cooperative Feed Co.) (Table 2). Fresh mushrooms were obtained from the Base Centre of Jilin Agricultural University, Changchun, China. Mushroom fruiting bodies were dried under sunlight for 72 h, and crushed into powder using a laboratory mill. Mushroom powder was mixed with the commercial diet as a daily intake of 8.5 g per individual. The nominated mushrooms' bioactive compounds are shown in Table 3.

**TABLE 2:** Chemical composition of experimental diets

Group	CP (%)	EE (%)	CF (%)	ADF (%)	NDF (%)	Ash (%)	CHO (%)
C+ and C−	19 ± 0.1^b^	4 ± 1^a^	38 ± 0.2^a^	36 ± 0.03^b^	46 ± 0.2^a^	7.2 ± 0.05^a^	68 ± 1^ab^
GLM	19 ± 1^b^	2.4 ± 0.5^a^	41 ± 3^a^	42 ± 0.1^a^	49 ± 0.5^a^	4.6 ± 1.07^a^	72 ± 1^a^
POM	24 ± 1^a^	4.1 ± 0.7^a^	37 ± 0.2^a^	42.89 ± 0.3^a^	42 ± 3^a^	6.3 ± 0.02^b^	64 ± 0.4^c^
PCM	22 ± 3^ab^	3.8 ± 0.6^a^	38 ± 0.1^a^	37 ± 1^b^	44 ± 0.3^ab^	6.6 ± 0.05^a^	64 ± 0.3^bc^
LEM	21 ± 2^ab^	3.8 ± 0.1^a^	39 ± 1^a^	41 ± 0.5^a^	46 ± 0.6^a^	6.2 ± 0.06^a^	67 ± 2^bc^
HMM	23 ± 0.9^ab^	3.4 ± 0.08^a^	37 ± 0.03^a^	36 ± 2^b^	45 ± 2^a^	6.9 ± 0.02^a^	65 ± 0.9^bc^
*p*‐Value	.073	.371	.295	.002	.141	.010	.013

*Note*: Values represented means ± standard deviation. Different letters represent a significant difference in Tukey test at *p* < .05.

Abbreviations: ADF, acid detergent fiber; C−, negative control; C+, positive control; CF, crude fiber; CHO, carbohydrate; CP, crude protein; EE, ether extract; GLM, *Ganoderma lucidum* mushroom; HMM, *Hypsizigus marmoreus* mushroom; LEM, *Lentinus edodes* mushroom; NDF, neutral detergent fiber; PCM, *Pleurotus citrinopileatus* mushroom; POM, *Pleurotus ostreatus* mushroom.

**TABLE 3:** Mushrooms' bioactive compounds (percentage of control)

Bioactive compound	C	GLM	POM	PCM	LEM	HMM
Agmatine	33	29	73	80	446	829
Stachyose	40	357	40	73	27	25
Sphingosine	31	94	18	23	68	621
Pyridoxine	70	247	32	112	25	21
Linolenic acid	68	104	82	99	145	143
Alanine	34	126	116	59	161	123

Abbreviations: C, control diet; GLM, *Ganoderma lucidum* mushroom; HMM, *Hypsizigus marmoreus* mushroom; LEM, *Lentinus edodes* mushroom; PCM, *Pleurotus citrinopileatus* mushroom; POM, *Pleurotus ostreatus* mushroom.

### 2.4 Liquid chromatography‐mass spectrometry (LC–MS)

#### 2.4.1 Sample preparation

Fifty milligrams of mushroom sample was mixed with 1 ml of the mixture (methanol:acetonitrile:water, 2:2:1). The sample was put into a multi‐tissue grinder (frequency 60 Hz, 4 min) for tissue fragmentation, and then ultrasonicated for 10 min and then it was kept in the refrigerator for 1 h. the sample was centrifuged at 4°C for 15 min at 10,000 *g*. Seven hundred microliters of supernatant was put in a vacuum freeze dryer until it evaporated. The solution was resuscitated with 500 μl acetonitrile water (1:1) for 30 s and ultrasonicated for 10 min. The centrifugation was performed at 4°C for 15 min at 10,000 *g*. A volume of 50 μl of supernatant was put into the injection bottle and detected by LC–MS.

#### 2.4.2 Liquid phase conditions

We used a chromatographic column (Waters ACQUITY UPLC BEH Amide 1.7 μm, 2.1 mm × 100 mm).

#### 2.4.3 Mobile phase

Phase A is ultrapure water containing 25 mM ammonium acetate and 25 mm ammonia, and phase B is acetonitrile. Current Speed 5 ml/min, column temperature 40°C, injection volume 2 μl.

#### 2.4.4 Liquid phase elution gradient

A gradient elution high‐performance liquid chromatographic method is described in Table 4.

**TABLE 4:** Liquid phase elution gradient

Time	A%	B%	Flow rate (ml/min)
0	5	95	0.5
0.5	5	95	0.5
7	35	65	0.5
8	60	40	0.5
9	60	40	0.5
9.1	5	95	0.5
12	5	95	0.5

#### 2.4.5 Mass spectrometry conditions

Temperature of EFI ion source was 65°C. MS voltage was 5500 V (positive ion) and was 4500 V (negative ion). Declustering voltage DP was 6 0 V Ion source gas: gas 1 was 60 psi, gas 2 was 60 psi, and curtain gas (cur) was 30 psi.

### 2.5 DNA extraction, polymerase chain reaction amplification and high‐throughput sequencing

Next‐generation sequencing (NGS), including library preparations, was conducted at Genewiz, Inc. using an Illumina MiSeq (Illumina). DNA (30–50 ng) was extracted using TIANGEN DP336 genomic‐DNA extraction kits (TIANGEN Biotech [Beijing] Co. Ltd.) and quantified with a Qubit 2.0 Fluorometer (Invitrogen). To generate amplicons (400–450 bp), the MetaVx Library Preparation Kit (Genewiz) was used. For each 40 ng sample of DNA, V3, V4, and V5 hypervariable regions of prokaryotic 16S ribosomal RNA (rRNA) were selected for generating amplicons, following taxonomic analysis. Genewiz has designed a panel of proprietary primers aimed at relatively conserved regions bordering the V3, V4, and V5 hypervariable regions of bacteria and Archaea16S rDNA (if eukaryotic DNA was contaminated, only the V3 and V4 regions were amplified). V3 and V4 regions were amplified using forward primers containing the sequence CCTACGGRRBGCASCAGKVRVGAAT and reverse primers containing the sequence GGACTACNVGGGTWTCTAATCC, the V4 and V5 regions were amplified using forward primers containing the sequence GTGYCAGCMGCCGCGGTAA and reverse primers containing the sequence CTTGTGCGGKCCCCCGYCAATTC. First‐round polymerase chain reaction (PCR) products were used as templates for second‐round amplicon enrichment PCR. At the same time, indexed adapters were added to the ends of the 16S rDNA amplicons to generate indexed libraries for downstream NGS on the Illumina MiSeq according to Quast et al. (2013).

DNA libraries were validated using an Agilent 2100 Bioanalyzer (Agilent Technologies), quantified by Qubit 2.0 Fluorometer (Invitrogen), multiplexed and loaded onto the Illumina MiSeq as per manufacturer's instructions. NGS was performed using a 2 × 300 paired‐end (PE) configuration (Li, Hu, et al., 2017; Li, Wang, et al., 2017). Image analysis was conducted and base calling with the MiSeq Control Software (MCS) embedded in the MiSeq instrument (Yilmaz et al., 2014). The amplicon sequence data were deposited with the National Center for Biotechnology Information (Accession Nos. SRR2579284 and ERS2011824).

### 2.6 Sequence analysis

Quantitative Insights into Microbial Ecology (QIIME) data analysis software were used to analyze 16S rRNA data (Caporaso et al., 2010). Quality filtering was performed on raw sequences according to Bokulich et al. (2012), as well as on joined sequences. Any sequence that was not <200 bp, with no ambiguous bases and a mean quality score ≥20 was discarded. Forward and reverse reads were joined and assigned to samples based on barcode and truncated by cutting off the barcode and primer sequence. The sequences were compared with the reference database (Ribosomal Database Project [RDP] Gold database) using the UCHIME algorithm (Edgar et al., 2011) to detect chimeric sequences that were removed. Only effective sequences were used in the final analysis. Sequences were grouped into operational taxonomic units (OTUs) and pre‐clustered at 97% sequence identity using the clustering program VSEARCH version 1.9.6 (Rognes et al., 2016) against the SILVA 119 database. The RDP classifier was used to assign taxonomic categories to all the OTUs at a confidence threshold of 0.8, according to Crawford et al. (2009). The RDP classifier uses the SILVA 119 database, which predicts taxonomic categories to the species level. Sequences were rarefied prior to calculation of alpha and beta diversity statistics. Alpha diversity indices were calculated in QIIME from rarefied samples using the *Shannon* index for diversity and the *Chao*1 index for richness (Chao, 1984; Chao & Lee, 1992). Beta diversity was calculated using weighted and unweighted UniFrac and principal component analysis (Bamberger & Lowe, 1988). An unweighted Pair Group Method with Arithmetic mean (UPGMA) tree from beta diversity distance matrix was built.

### 2.7 Statistical analysis

Based on the beta diversity distance matrix and on environmental factor data, canonical correspondence analysis (CCA) between RFPs and BCC was integrated by the R‐language software application (Team, 2018). All data were analyzed by one‐way (mushroom type) analysis of variance (ANOVA) and were performed using SPSS‐software, version 11.5 (SPSS, Version 11.5.0; SPSS Inc.). Results were expressed as Mean ± SD. Tukey's contrasts were used to test the significance level for the effects of mushroom types, with *p* < .05 indicating significant difference.

## 3 RESULTS

### 3.1 Feed intake, plasma glucose level, and body weight

There were significant differences in feed intake between PCM and C−, and between POM and C+ (*p <* .05) (Table 5). All mushroom treatments showed a significant difference compared with C+ except GLM treatments in plasma glucose level, whereas the mushrooms treatments showed a gradually improved plasma glucose level till 45 days of experimentation (*p <* .05). LEM and HMM showed less significant difference compared with C− in plasma glucose level (*p <* .05).

**TABLE 5:** Feed intake and plasma glucose level

Group	Feed intake (g/day)	Plasma glucose (mmol)	
		Before treatment	After treatment
C−	37 ± 3^bc^	6 ± 0.4^b^	5 ± 0.3^d^
C+	44 ± 2^ab^	26 ± 6^a^	23 ± 6^a^
GLM	43 ± 1^ab^	28 ± 5^a^	21 ± 4^ab^
POM	33 ± 12^c^	28 ± 4^a^	16 ± 3^bc^
PCM	47 ± 2^a^	26 ± 2^a^	15 ± 5^c^
LEM	40 ± 3^abc^	24 ± 8^a^	11 ± 5^c^
HMM	43 ± 2^ab^	25 ± 6^a^	12 ± 2^c^
*p*‐Value	.005	.001	.001

*Note*: Values represented means ± standard deviation. Different letters represent a significant difference in Tukey test at *p* < .05.

Abbreviations: C−, negative control; C+, positive control; GLM, *Ganoderma lucidum* mushroom; HMM, *Hypsizigus marmoreus* mushroom; LEM, *Lentinus edodes* mushroom; PCM, *Pleurotus citrinopileatus* mushroom; POM, *Pleurotus ostreatus* mushroom.

Mushroom treatments showed a significant difference compared with C− in body weight (*p <* .05) (Table 6). HMM treatment showed a significant difference compared with control (C− and C+) in liver weight (*p <* .05).

**TABLE 6:** Body weight indices and plasma glucose levels in alloxan‐induced diabetic rats received normal or mushroom diets

Treatments	Body weight (g)			
	First	Second	Third	Final
C−	253 ± 5^a^	338 ± 22^a^	370 ± 21^a^	411 ± 31^a^
C+	245 ± 4^ab^	267 ± 17^b^	283 ± 20^b^	234 ± 45^b^
GLM	248 ± 7^ab^	2790 ± 31^b^	296 ± 47^b^	246 ± 74^b^
POM	240 ± 6^b^	266 ± 21^b^	268 ± 33^b^	220 ± 43^b^
PCM	250 ± 5^a^	266 ± 20^b^	284 ± 20^b^	232 ± 48^b^
LEM	247 ± 8^ab^	268 ± 11^b^	276 ± 22^b^	221 ± 42^b^
HMM	244 ± 5^ab^	267 ± 12^b^	281 ± 23^b^	187 ± 39^b^
*p*‐Value	.047	.001	.001	.001

*Note*: Values represented means ± standard deviation. Different letters represent a significant difference in Duncan test at *p* < .05.

Abbreviations: C−, negative control; C+, positive control; GLM, *Ganoderma lucidum* mushroom; HMM, *Hypsizigus marmoreus* mushroom; LEM, *Lentinus edodes* mushroom; PCM, *Pleurotus citrinopileatus* mushroom; POM, *Pleurotus ostreatus* mushroom.

### 3.2 Untargeted molecules analysis by LC/MS

A wide variety of molecules have been identified in diet and mushroom samples, the molecules level was calculated as a percent of the quality control (QC) value, a percent over 100 was nominated as a possible effective molecule. Among them, few molecules have been discussed in this study based on the available literature regarding to the effect on diabetes (Table 7).

**TABLE 7:** Untargeted molecule analysis by LC/MS

SN	Molecule name	Response (%)					
		Diet	GLM	POM	PCM	LEM	HMM
1	(−)‐Riboflavin	175	150	6	136	72	69
2	1,3‐Diaminopropane	63	258	34	59	3	3
3	11‐Keto‐beta‐boswellic acid	51	3	30	87	374	360
4	4‐Methoxyphenylacetic acid	198	55	120	126	32	29
5	Acetylcholine	61	63	297	51	16	14
6	Adenine	125	143	150	65	9	9
7	Adenosine	162	125	61	145	16	16
8	Ajmalicine	156	69	44	119	94	89
9	Allopurinol	215	43	35	163	63	60
10	Betaine	80	86	118	96	117	122
11	Choline	101	61	198	114	35	31
12	Cytidine 5′‐diphosphocholine (CDP‐choline)	172	7	125	179	3	4
13	Cytosine	40	331	15	41	62	64
14	Dehydroascorbic acid (oxidized vitamin C)	19	13	157	34	13	13
15	Diaveridine	90	233	32	56	430	432
16	Dimethylglycine	110	43	176	125	39	36
17	dl‐2‐Aminoadipic acid	34	29	171	23	25	24
18	d‐Mannitol	131	152	142	42	12	10
19	Dopamine	195	8	120	216	15	15
20	d‐Ornithine	117	75	376	90	31	13
21	Edaravone	142	87	80	150	95	81
22	Eicosapentaenoic acid	30	14	7.55	18	389	348
23	Ephedrine	307	20	95	22	17	13
24	gamma‐l‐Glutamyl‐l‐valine	115	98	56	80	129	113
25	Glucosamine	119	175	82	146	28	24
26	Glutathione disulfide	208	84	228	174	29	20
27	Gly‐Val	228	111	43	139	112	36
28	Guanosine	139	199	53	160	28	24
29	Gutathione	76	159	73	146	23	31
30	Harmane	120	98	72	112	82	80
31	His‐Pro	247	28	106	149	46	45
32	Inosine	187	77	51	145	65	63
33	Isopentenyladenosine	342	103	56	175	48	51
34	Isosorbide	121	160	137	32	15	13
35	l‐Abrine	141	58	30	305	53	47
36	l‐Alanine	146	61	154	117	30	29
37	Lanosterol	14	1	213	0.94	0.28	1
38	l‐Arginine	120	110	98	139	132	109
39	l‐Asparagine	162	53	54	143	16	10
40	l‐Aspartate	179	29	67	166	75	58
41	l‐Carnitine	83	87	112	135	51	47
42	l‐Citrulline	317	72	115	35	110	110
43	l‐Glutamine	107	157	67	63	38	30
44	l‐Histidine	334	43	279	168	13	9
45	Linoleic acid	87	104	77	100	127	116
46	Linoleoyl ethanolamide	206	70	151	111	46	53
47	l‐Methionine	443	19	16	99	11	12
48	l‐Tyrosine	215	20	114	232	15	14
49	l‐Valine	46	13	193	26	28	28
50	Maltopentaose	39	315	31	81	25	17
51	Maltotriose	38	301	50	174	19	17
52	Meperidine	76	20	395	30	14	23
53	Miltefosine	95	86	153	185	94	95
54	*N*,*N*‐dimethylsphingosine	165	100	156	103	276	276
55	*N*‐acetyl‐d‐glucosamine	17	534	14	30	15	13
56	*N*‐acetyl‐l‐tyrosine	95	55	261	165	90	74
57	Nalidixic acid	44	170	15	53	215	215
58	NG,NG‐dimethyl‐l‐arginine (ADMA)	207	81	177	185	39	14
59	Nicotinamide	157	141	75	181	4	4
60	Oleic acid	56	78	88	86	135	146
61	Pantothenate	63	87	288	138	5	5
62	Phenyllactic acid	197	61	112	131	27	25
63	Phytosphingosine	31	94	18	23	689	621
64	*p*‐Phenylenediamine	95	102	97	99	99	98
65	Pseudouridine	76	204	44	75	46	37
66	Pyridoxine	70	247	32	112	25	21
67	Ribitol	25	190	21	38	184	163
68	Serotonin	174	13	4	266	15	16
69	S‐Methyl‐5′‐thioadenosine	381	151	0.82	54	0.75	0.66
70	Stachyose	40	357	40	73	27	25
71	Tetraethylene glycol	27	36	37	21	13	15
72	Tetramisole	96	210	71	739	46	28
73	Thymine	135	71	96	250	71	71
74	*trans*‐Vaccenic acid	60	99	90	89	151	150
75	Triethylene glycol	22.10	61	36	32	19	17
76	Trimethoprim	79	302	37	150	36	29
77	Tropine	88	114	51.31	131	224	193
78	Tyramine	211	46	119	138	28	22
79	Uracil	137	133	42	136	22	21
80	Uridine	144	124	45	129	24.02	22.49
81	Xanthosine	447	19	15	45	2.68	2.55

Abbreviations: GLM, *Ganoderma lucidum* mushroom; HMM, *Hypsizigus marmoreus* mushroom; LEM, *Lentinus edodes* mushroom; PCM, *Pleurotus citrinopileatus* mushroom; POM, *Pleurotus ostreatus* mushroom.

### 3.3 Bacterial diversity and community structure

#### 3.3.1 DNA sequencing

All colon content samples (*n* = 36) produced 415,315 original raw sequences. A total of 145,965 high‐quality bacterial sequences (average 4054 sequences per sample) were obtained after sequence cleanup (Table 8).

**TABLE 8:** OTU classification and classification status identification results statistical table

Group	Phylum	Class	Order	Family	Genus	Species	Unclassified	Total
C−	7261	7261	7260	5713	2312	202	7	30,016
C+	2121	2121	2121	2076	1554	36	2	10,031
GLM	4279	4279	4278	3483	1223	111	4	17,657
POM	4699	4699	4698	4029	1519	108	3	19,755
PCM	5333	5333	5333	4419	1754	155	7	22,334
LEM	5477	5477	5477	4579	1867	169	4	23,050
HMM	5510	5510	5510	4609	1844	134	5	23,122
Total	34,680	34,680	34,677	28,908	12,073	915	32	145,965

*Note*: “Phylum”, “Class”, “Order”, “Family”, “Genus” and “Species” respectively correspond to the number of OTUs that can be classified into doors, classes, orders, families, genera, and species in each sample, and “Unclassified” refers to the number of OTUs that failed to belong to any known taxon.

#### 3.3.2 OTU classification

Similarity among OTUs that were classified as belonging to different phylum, classes, orders, families, genus, and species (Table 8) based on 16S rRNA gene sequences revealed higher abundances for PCM, LEM, and HMM treatments at phylum and genus levels.

#### 3.3.3 Principle component analysis

**FIGURE 1:**
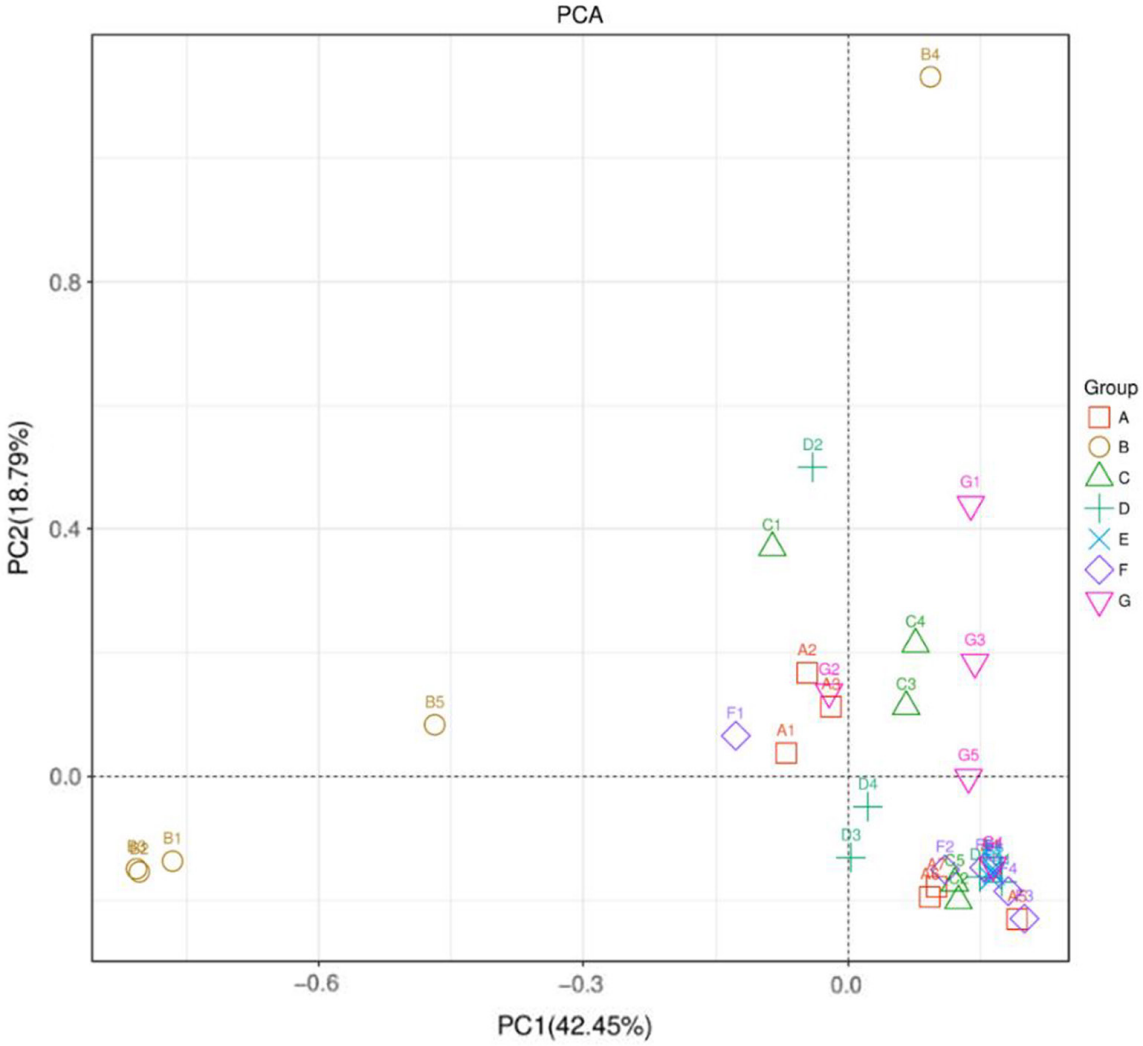
Principle component analysis (PCA) of profiling data from intestinal metabolome. (A, C−) Negative control; (B, C+) positive control; (C, GLM) *Ganoderma lucidum* mushroom; (D, POM) *Pleurotus ostreatus* mushroom; (E, PCM) *Pleurotus citrinopileatus* mushroom; (F, LEM) *Lentinus edodes* mushroom; (G, HMM) *Hypsizigus marmoreus* mushroom

Principle component analysis analysis revealed clear divisions among treatment groups. Group B (C+) was “significantly different” from all other treatments, expressing a clear effect of mushroom treatments (*p <* .05) (Figure 1). More similarity was observed among A (C−), C (GLM), and G (HMM) compared with D (POM), E (PCM), and F (LEM) while similarity between E (PCM) and F (LEM) was higher.

#### 3.3.4 Alpha diversity

Table 9 showed that indices of *ACE*, *Chao1*, *Shannon*, and *Simpson* were significantly affected by PCM and LEM treatments (*p* < .05), while ACE, Shannon and Simpson indexes were affected by HMM treatment (*p* < .01). Groups C+ and POM affected the Simpson index. All these four indices were lower in GLM treatment (*p* < .05).

**TABLE 9:** Effect of different dietary mushrooms on alpha diversity of gut microbiota communities in diabetic rats

Treatments	ACE	Chao 1	Shannon	Simpson
C−	1138 ± 321^ab^	1121 ± 330^ab^	7.30 ± 0.9^a^	0.96 ± 0.03^a^
C+	465 ± 117^b^	448 ± 112^b^	4.60 ± 0.9^b^	0.78 ± 0.1^b^
GLM	1018 ± 351^ab^	993 ± 346^ab^	6.54 ± 1^ab^	0.92 ± 0.08^a^
POM	1092 ± 627^ab^	1042 ± 591^ab^	6.59 ± 1^ab^	0.93 ± 0.05^a^
PCM	1598 ± 352^a^	1542 ± 349^a^	7.75 ± 0.3^a^	0.97 ± 0.01^a^
LEM	1303 ± 474^a^	1249 ± 443^a^	7.13 ± 0.8^a^	0.96 ± 0.02^a^
HMM	1319 ± 485^a^	1239 ± 452^ab^	6.82 ± 1^a^	0.94 ± 0.03^a^
*p*‐Value	.012	.012	.003	.002

*Note*: Values represented means ± standard deviation. Different letters represent a significant difference in Duncan test at *p* < .05. The first column in the table is groups, and the subsequent columns correspond to the calculation results of the diversity index of *Chao1*, *ACE*, *Shannon*, and *Simpson* and so on for each sample at the same sequencing depth.

Abbreviations: C−, negative control; C+, positive control; GLM, *Ganoderma lucidum* mushroom; HMM, *Hypsizigus marmoreus* mushroom; LEM, *Lentinus edodes* mushroom; PCM, *Pleurotus citrinopileatus* mushroom; POM, *Pleurotus ostreatus* mushroom.

#### 3.3.5 Bacterial community composition (BCC)

**FIGURE 2:**
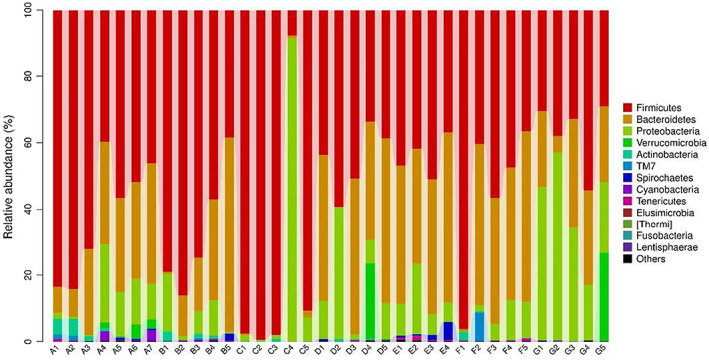
Microbial populations at phylum level (%). *Note*: The abscissa depicts the sample name, and the ordinate shows the number of microbial phyla. A: negative control, B: positive control, C: GLM, D: POM, E: PCM, F: LEM, and G: HMM

At phylum level, C+ treatment showed higher *Firmicutes* but lower *Bacteroidetes* abundances. In contrast, HMM treatment showed lower *Firmicutes* abundance. C+ treatment PCM treatment showed higher *Bacteroidetes* abundance. LEM treatment showed lower *Proteobacteri* and *Verrucomicrobi* abundances. In contrast, HMM treatment showed higher *Proteobacteri* and *Verrucomicrobi* abundances (Table 10 and Figure 2).

**TABLE 10:** Composition of gut microbiota communities at phylum level (%)

Treatments	Firmicutes	Bacteroidetes	Proteobacteria	Verrucomicrobia
C−	62 ± 17^ab^	23 ± 11^ab^	9 ± 8^a^	1 ± 1^a^
C+	78 ± 39^a^	0.69 ± 0.8^b^	20 ± 39^a^	0.019 ± 0.02^a^
GLM	67 ± 19^ab^	23 ± 22^ab^	7.31 ± 6^a^	0.036 ± 0.05^a^
POM	45 ± 1^b^	35 ± 20^a^	14 ± 14^a^	4.6 ± 10^a^
PCM	44 ± 6^b^	42 ± 6^a^	10 ± 72^a^	0.011 ± 0.008^a^
LEM	55 ± 24^ab^	35 ± 2^a^	6 ± 5^a^	0.0035 ± .007^a^
HMM	36 ± 1^b^	22 ± 1^ab^	35 ± 16^a^	5 ± 11^a^
*p*‐Value	.05	.006	.169	.577

*Note*: Values represented means ± standard deviation. Different letters represent a significant difference in Duncan test at *p* < .05.

Abbreviations: C−, negative control; C+, positive control; GLM, *Ganoderma lucidum* mushroom; HMM, *Hypsizigus marmoreus* mushroom; LEM, *Lentinus edodes* mushroom; PCM, *Pleurotus citrinopileatus* mushroom; POM, *Pleurotus ostreatus* mushroom.

**FIGURE 3:**
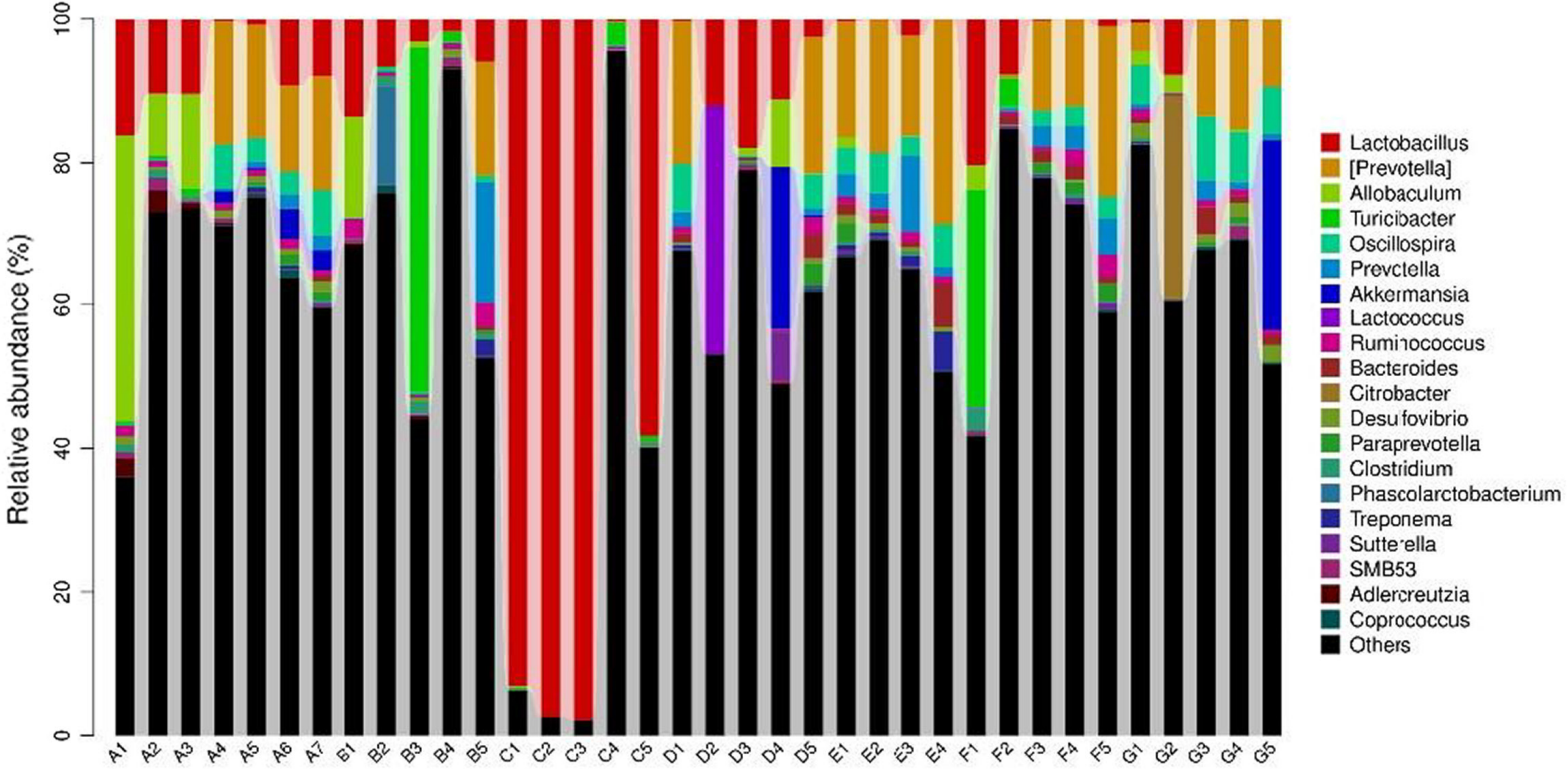
Microbial populations at genus levels (%). *Note*: The abscissa depicts the sample name, and the ordinate shows the number of microbial genera. A: negative control, B: positive control, C: GLM, D: POM, E: PCM, F: LEM, and G: HMM

At genus level, Peptostreptococcaceae abundance was higher in C− and GLM treatments. Enterobacteriaceae abundance was higher in C+, POM, and HMM. *Allobaculum* abundance was high only in C− treatment (Table 11 and Figure 3).

**TABLE 11:** Composition of gut microbiota communities at genus level (%)

Genus	Treatments							*p*‐Value
	C−	C+	GLM	POM	PCM	LEM	HMM	
Peptostreptococcaceae	0.15 ± 0.2^a^	0.06 ± 0.101^a^	0.18 ± 0.2^a^	0.03 ± 0.04^a^	0.01 ± 0.001^a^	0.02 ± 0.03^a^	0.06 ± 0.09^a^	.296
Enterobacteriaceae	0.01 ± 0.001^a^	0.2 ± 0.3^a^	0.07 ± 0.06^a^	0.07 ± 0.1^a^	0.01 ± 0.01^a^	0.01 ± 0.005^a^	0.15 ± 0.1^a^	.427
Desulfovibrionaceae	0.07 ± 0.07a	0.01 ± 0.001^a^	0.01 ± 0.001^a^	0.04 ± 0.05^a^	0.09 ± 0.07^a^	0.05 ± 0.05^a^	0.06 ± 0.05^a^	.117
Ruminococcaceae	0.04 ± 0.03^bc^	0.01 ± 0.001^c^	0.03 ± 0.03^bc^	0.05 ± 0.03^bc^	0.09 ± 0.03^a^	0.07 ± 0.05^ab^	0.03 ± 0.01^bc^	.008
*Allobaculum*	0.09 ± 0.01^a^	0.01 ± 0.001^a^	0.03 ± 0.06^a^	0.02 ± 0.04^a^	0.01 ± 0.007^a^	0.01 ± 0.01^a^	0.01 ± 0.01^a^	.376
*Turicibacter*	0.01 ± 0.003^a^	0.01 ± 0.01^a^	0.10 ± 0.02^a^	0.01 ± 0.001^a^	0.01 ± 0.001^a^	0.07 ± 0.1^a^	0.01 ± 0.001^a^	.464
*Oscillospira*	0.03 ± 0.02^abc^	0.01 ± 0.001^c^	0.01 ± 0.003^c^	0.02 ± 0.03b^c^	0.04 ± 0.015^ab^	0.02 ± 0.01^bc^	0.06 ± 0.03^a^	.005
Lachnospiraceae	0.01 ± 0.01^bc^	0.01 ± 0.001^c^	0.01 ± 0.01^bc^	0.03 ± 0.03^abc^	0.05 ± 0.03^a^	0.03 ± 0.003^ab^	0.02 ± 0.01^bc^	.032
*Prevotella*	0.01 ± 0.009^a^	0.01 ± 0.001^a^	0.03 ± 0.07^a^	0.01 ± 0.009^a^	0.04 ± 0.04^a^	0.02 ± 0.02^a^	0.01 ± 0.001^a^	.374
*Akkermansia*	0.01 ± 0.01^a^	0.01 ± 0.001^a^	0.01 ± 0.001^a^	0.05 ± 0.1^a^	0.01 ± 0.001^a^	0.01 ± 0.001^a^	0.05 ± 0.1^a^	.577
Clostridiaceae	0.02 ± 0.02^a^	0.02 ± 0.027^a^	0.03 ± 0.02a	0.01 ± 0.002^a^	0.00 ± 0.001^a^	0.03 ± 0.06^a^	0.01 ± 0.01^a^	.664
*Ruminococcus*	0.01 ± 0.005^ab^	0.01 ± 0.002^b^	0.02 ± 0.01^a^	0.01 ± 0.001^ab^	0.01 ± 0.003^ab^	0.01 ± 0.003^ab^	0.01 ± 0.004^ab^	.219
*Bacteroides*	0.01 ± 0.003^ab^	0.01 ± 0.001^b^	0.01 ± 0.002^ab^	0.01 ± 0.01^ab^	0.02 ± 0.02^a^	0.01 ± 0.008^ab^	0.01 ± 0.01a^b^	.037

*Note*: Values represented means ± standard deviation. Different letters represent a significant difference in Duncan test at *p* < .05.

Abbreviations: C−, negative control; C+, positive control; GLM, *Ganoderma lucidum* mushroom; HMM, *Hypsizigus marmoreus* mushroom; LEM, *Lentinus edodes* mushroom; PCM, *Pleurotus citrinopileatus* mushroom; POM, *Pleurotus ostreatus* mushroom.

## 4 DISCUSSION

Mushrooms have antihyperglycemic effects on diabetic individuals. LEM and HMM treatments showed lower plasma glucose levels. LEM and PCM changed the *ACE*, *Chao1*, *Shannon*, and *Simpson* microbial indices significantly. Dietary supplementation of mushrooms reduced plasma glucose level directly due to mushrooms' bioactive compounds (agmatine, sphingosine, pyridoxine, linolenic, and alanine) and indirectly through oligosaccharide (stachyose) and gut microbiota modulation. Thus, LEM and HMM can be used as healthy food ingredients to improve gut microbiome composition in diabetic subjects.

Agmatine shows an antihyperglycemic effect through increasing insulin secretion and glucose uptake in muscles, and preventing hepatic gluconeogenesis via β‐endorphin secretion enhancement (Su et al., 2009). Since agmatine simulates insulin secretion via inhibiting the ATP‐sensitive potassium (KATP) channels in β‐islet cells (Chang et al., 2010; Malaisse et al., 1989; Naoki & Fujiwara, 2019; Nissim et al., 2006, 2014; Shepherd et al., 2012; Su et al., 2008). Such inhibited KATP channel elevates the ATP/ADP ratio, leading to K^+^ accumulation. This cell depolarization simulates a voltage‐gated Ca^2+^ channel activity, resulting in Ca^2+^ influx and consequent insulin secretion (Velasco et al., 2016). In this study, mushroom treatments (LEM and HMM) showed the lowest blood glucose level, which can be explained by the high content of agmatine in LEM and HMM mushrooms. At the intestinal microbiota level, intestinal bacteria altered insulin secretion via converting arginine to agmatine. Such conversion can be catabolized by bacterial acid‐resistant mechanisms, such as *Escherichia coli* and *Enterococcus faecalis* (Naoki & Fujiwara, 2019). In our study, LEM treatment showed a high level of *Bacteriodetes* and *Firmicutes* phyla, whereas the HMM treatment showed a high level of *Proteobacteria*. That LEM and HMM mushrooms could manipulate the microbiota composition leading to an increased level of secreted insulin.

Sphingosine (PHS) has a potential therapeutic effect on type II diabetes (Nagasawa et al., 2018). Since PHS activates omega‐3 fatty acid receptor (GPR120) mediating potent insulin‐sensitizing effects. Such activation promotes incretin GLP‐1 secretion, which is notable for having an effects on an anti‐metabolic syndrome (Nagasawa et al., 2018). In this study, the HMM mushroom showed the highest level of PHS bioactive compound along with lower blood glucose levels (Rudd et al., 2020). In addition, the HMM mushroom showed the highest level of sphingosine along with lower blood glucose levels.

Pyridoxine decreases insulin resistance via scavenging the pathogenic reactive carbonyl species (Haus & Thyfault, 2018). Such molecule damages insulin protein via covalent modification of some structural amino acids, as well as, via the formation of adducts with phospholipids and DNA (Haus & Thyfault, 2018). In the current study, the GLM mushroom showed the highest level of pyridoxine bioactive compound along with lower blood glucose levels.

Nutritive acids could have a controversial effect on diabetic individuals. For instance, α‐linolenic acid is a source for the generated oxylipins. Such molecules are lipid mediators affecting type 1 diabetes (Buckner et al., 2021). In our study, the LEM and HMM mushrooms showed the highest level of linolenic acid along with lower blood glucose levels. However, alanine may induce hyperglycemia in diabetic individuals. Since alanine aminotransferases increased levels are marked in diabetes hepatic cells (Okun et al., 2021). In the current study, the LEM mushroom showed the highest level of alanine along with lower blood glucose levels. The amino acids and fatty acids classes should be considered in the research of diabetic individuals' diet.

In literature, the mushrooms showed different polysaccharides and their effect on diabetes, for example, *G. lucidum* (polyheterosaccharides); *P. ostreatus*, (polyheterosaccharides); *P. citrinopileatus* (acid polysaccharide); *L. edodes* (glucan, heteropolysaccharides); and *H. marmoreus* (glycoprotein). *Chlorella pyrenoidosa* polysaccharides with a low molecular weight (>3000 Da) showed an hypolipidemic effect in rat (Agunloye & Oboh, 2022; Hossain et al., 2021; Jayasuriya et al., 2015; Qiu et al., 2022). *Chisandra sphenanthera* polysaccharide (191.18 kD) showed antidiabetic effect in rats with type 2 diabetes (Niu et al., 2017). *Dendrobium officinale* leaf polysaccharides of different molecular weights were orally administered daily at 200 mg/kg/day, this level alleviated type II diabetes in an adult mouse (Fang et al., 2022). Thus, polysaccharides have an effect on diabetes whether they are low or high molecular weights. Stachyose is a non‐reducing tetrasaccharide molecule which decreases the blood glucose level in alloxan‐induced diabetic rats (Zhang et al., 2004). In addition, stachyose adjusts blood lipid levels in diabetic individuals (Chen et al., 2019). In the current study, the GLM mushroom showed the highest level of stachyose bioactive compound along with lower blood glucose levels. At the intestinal microbiota level, stachyose as a functional oligosaccharide regulates the intestinal microflora balance. Such prebiotic shifts of gut microbiota including *Bifidobacterium* and *Lactobacillus* as they are two common genera affecting a host health (Liu, Jia, et al., 2018; Liu, Wang, et al., 2018). In this study, the LEM and HMM mushrooms showed the highest level of *Lactobacillus* genus along with lower blood glucose levels. LEM and HMM mushrooms could modulate blood glucose levels and intestinal microbiota in diabetic individuals.

Changes in the gut microbiota composition are associated with multiple chronic disease pathologies, such as type 2 diabetes mellitus (Tang et al., 2017). Dietary fiber intake protects against diabetes by lowering dietary glycemic (Anderson et al., 2009). For example, oyster and button mushrooms have hypoglycemic effects, which reduce the fasting blood glucose level (Shehata et al., 2010). *G. lucidum* extract reduces blood glucose and insulin levels in rats (Hikino et al., 1985). Dietary supplements of *I. bartlettii*, *Bifidobacterium longum*, and *B. cellulosilyticus* in combination with water‐soluble viscous fibers improve glucose homeostasis and dyslipidemia. Since gut microbiota affects insulin resistance by decreasing TNF‐ α level in plasma and improving fasting blood glucose level in mice fed a high‐fat diet (Chuang et al., 2012).

Mushroom intake increases lactic acid‐producing bacteria (*Lactobacillus*, *Lactococcus* and *Streptococcus*) and SCFA‐producing bacteria (*Allobaculum*, *Bifidobacterium* and *Ruminococcus*), which can be explained by the mushroom's fiber content (Takamitsu et al., 2018). Butyrate‐producing *R. inulinivorans* abundance is higher in healthy individuals than in T2D individuals (Tang et al., 2017). *F. prausnitzii* abundance is low in individuals with T2D (Karlsson et al., 2013; Qin et al., 2012). Insulin resistance is related to *B. wadsworthia* and *C. bolteae* abundances (Qin et al., 2012). The species *A. muciniphila*, *B. faecis*, *B. nordii*, *B. cellulosilyticus*, *B. pectinophilus*, *I. bartlettii*, *O. splanchnicus*, *D. longicatena*, and *R. inulinivorans* were negatively associated with insulin resistance or dyslipidemia (Brahe et al., 2015). *Bifidobacterium (B. longum*) abundance was higher in healthy individuals than in obese individuals and T2D (Karlsson et al., 2013). The more decrease in butyric acid production, the more decrease in *C. leptum* abundance (Wang et al., 2015). Butyrate has an anti‐inflammatory activity that could improve insulin resistance (Brahe et al., 2013). *F. prausnitzii* affects insulin sensitivity, which may be due to its ability to produce butyrate (Louis & Flint, 2009). In this study, *Allobaculum* was increased in positive control treatment, whereas the *Ruminococcus* was increased in GLM treatment.

Regarding the mushroom mix potence, as individual mushrooms showed a significant effect on blood glucose level that mushroom mix could provide more effective role in blood glucose control regarding the gathered bioactive compounds and their possible compatible roles. Future studies are required to investigate the potency of mushroom mix with more diabetes parameters to reveal the underlying mechanism on diabetes‐based long‐term treatment.

## 5 CONCLUSIONS

Dietary supplementation with mushrooms reduced the plasma glucose level and modulated gut microbiota in diabetic rats. Mushrooms showed a direct antihyperglycemic effect due to their content of agmatine, stachyose, phytosphingosine, and pyridoxine bioactive compounds. Mixed dietary mushrooms could develop a food ingredient with an effective and specific health functionality for diabetic individuals.

## CONFLICT OF INTEREST

None.

## Data Availability

The data that support the findings of this study are available on request from the corresponding author.
